# Supplementary material for: Genome surveillance by HUSH-mediated silencing of intronless mobile elements
Source: Nature. 2021 Nov 18;601(7893):440–5. doi: 10.1038/s41586-021-04228-1 (PMC8770142; doi:10.1038/s41586-021-04228-1)
Supplement: Supplementary file 1 — This file contains Supplementary Figs. 1, 2. [file 41586_2021_4228_MOESM1_ESM.pdf]

nature portfolio

https://doi.org/10.1038/s41586-021-04228-1

Supplementary information

Genome surveillance by HUSH-mediated silencing of intronless mobile elements

In the format provided by the authors and unedited

Genome surveillance by HUSH-mediated silencing of intronless mobile elements

Seczynska et al.

Supplementary Information

Supplementary Figure 1-2

Fig. 3e

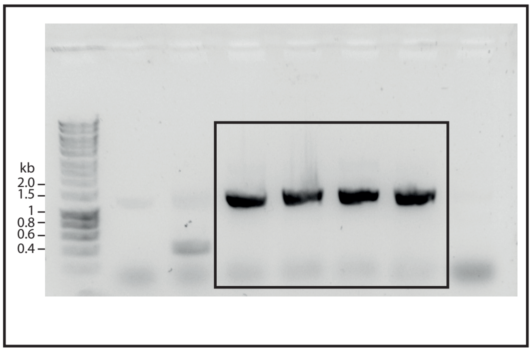

Extended data Fig 1b

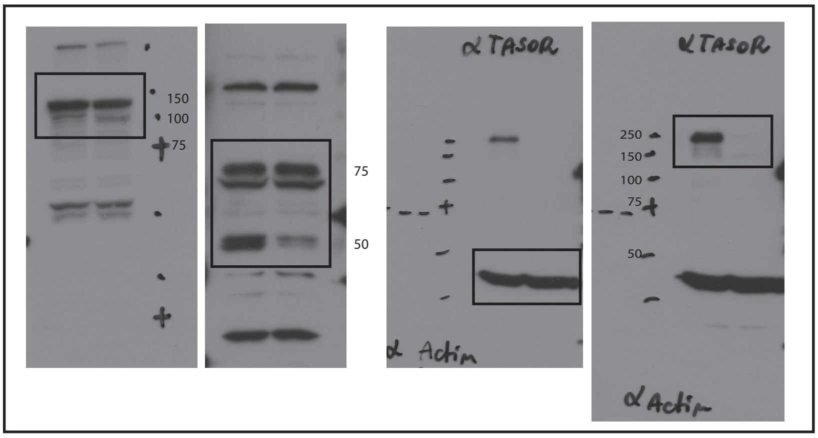

Extended data Fig. 1e

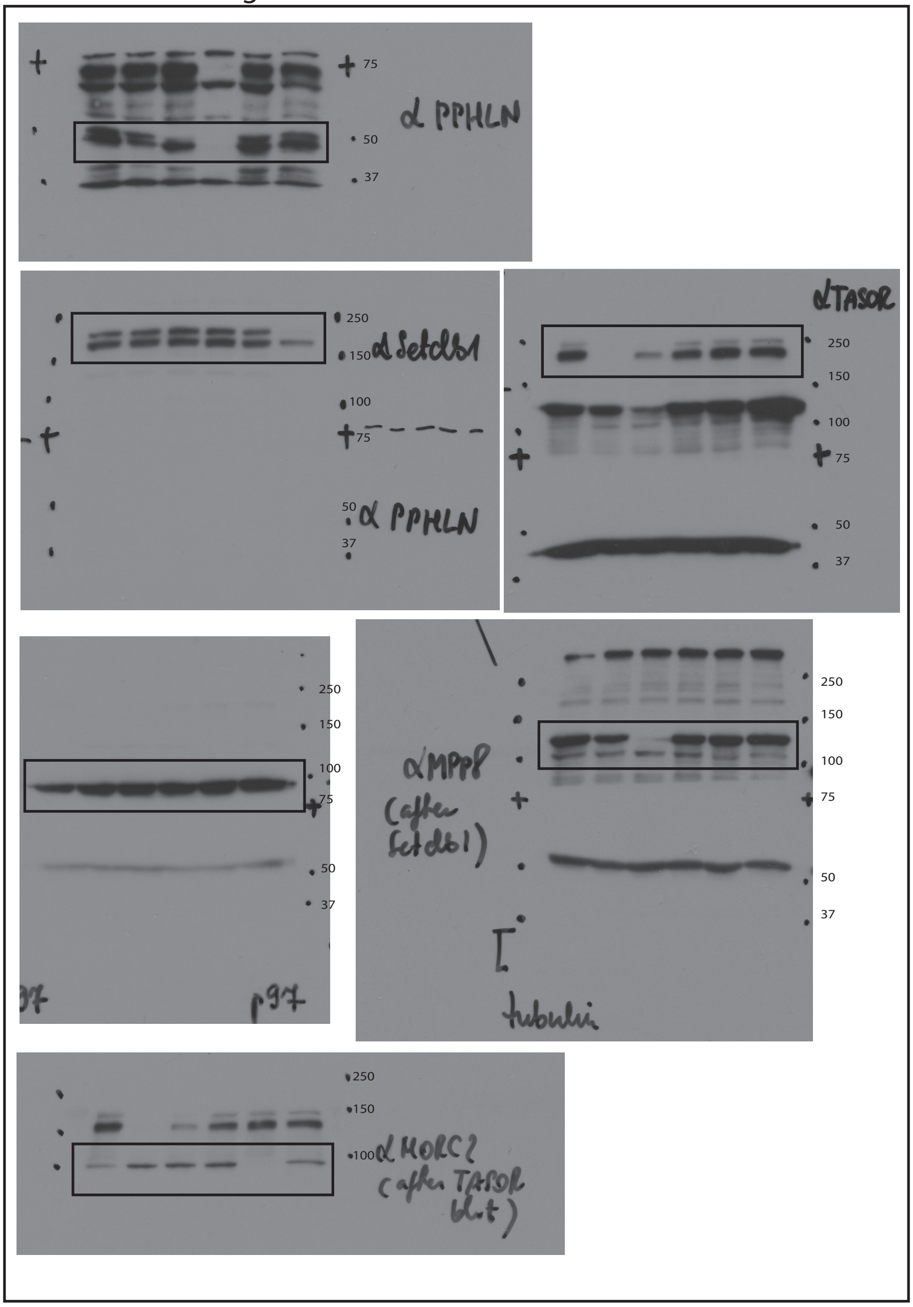

Extended data Fig. 1f

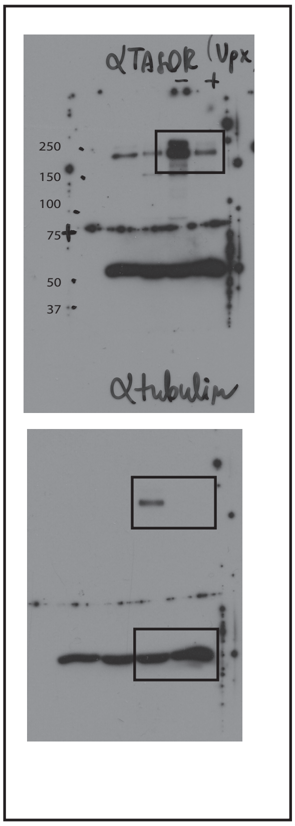

Extended data Fig. 1h
Extended data Fig. 1j
Extended data Fig. 4f

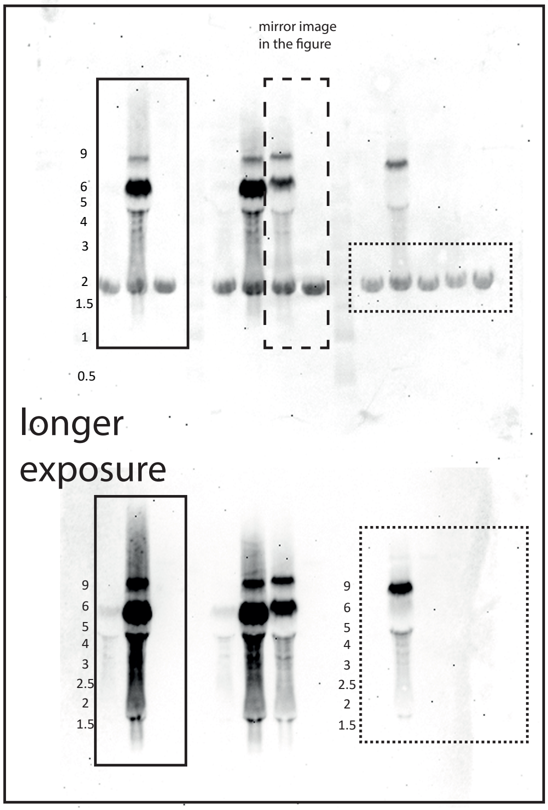

Extended data Fig. 2a

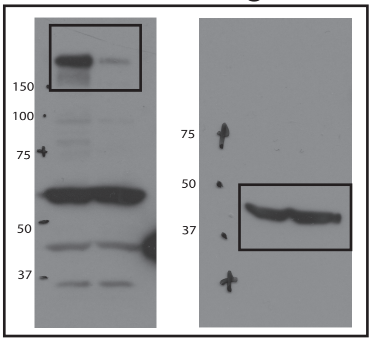

Extended data Fig.2k

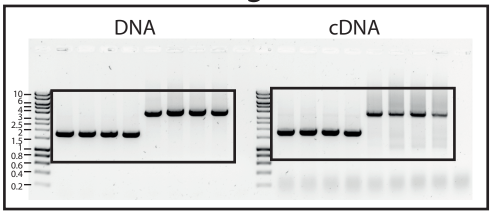

Extended data Fig. 4l

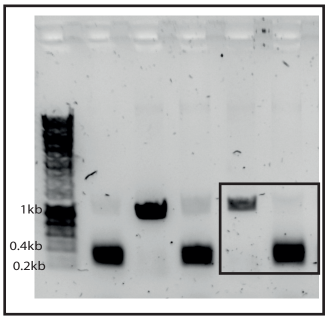

Extended data Fig.3g

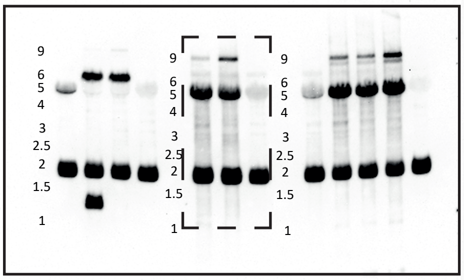

Extended data Fig.4c

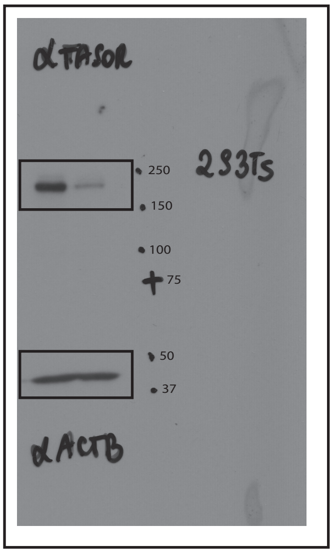

Extended data Fig. 4m

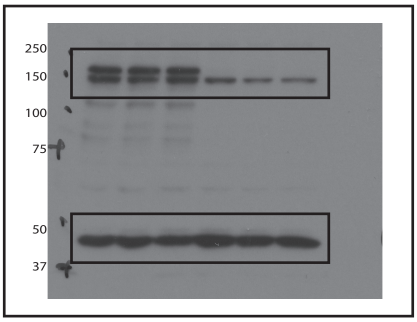

Extended data Fig. 4k

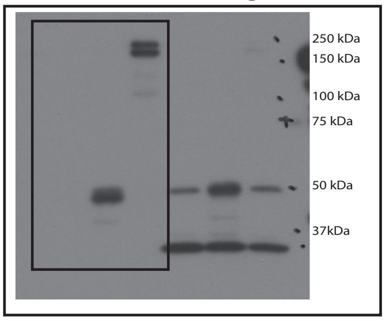

Extended data Fig. 5b
Extended data Fig. 7h

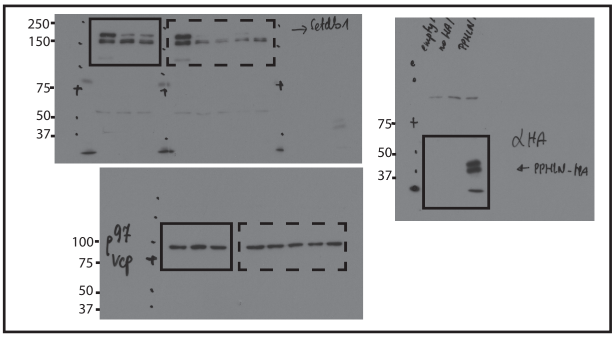

Extended data Fig. 8b

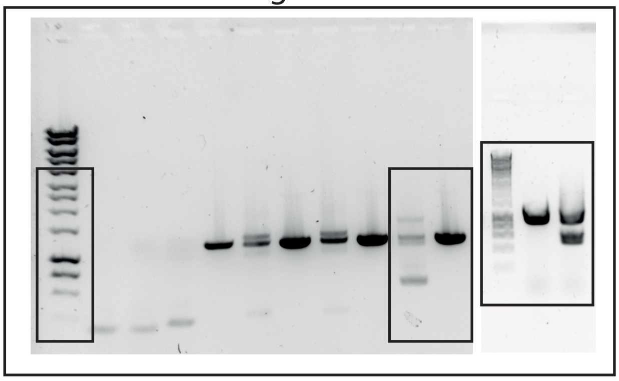

Extended data Fig. 6b

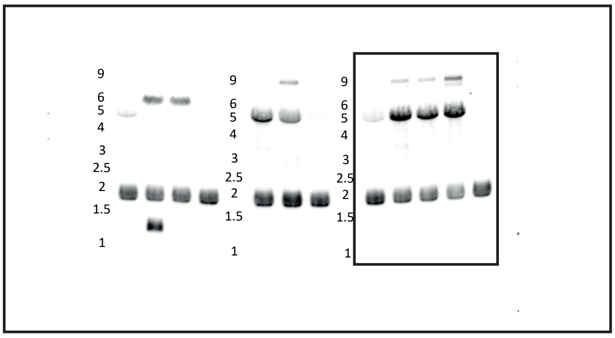

Extended data Fig. 8c

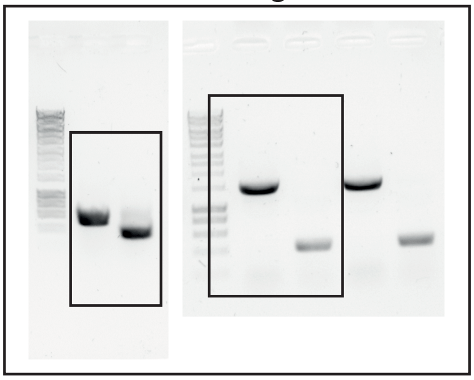

Extended data Fig. 6f

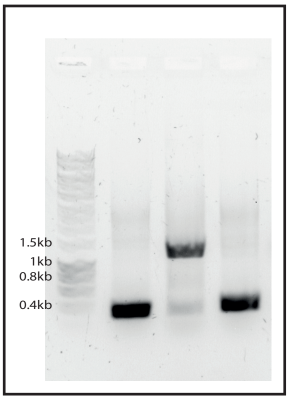

Extended data Fig. 7d

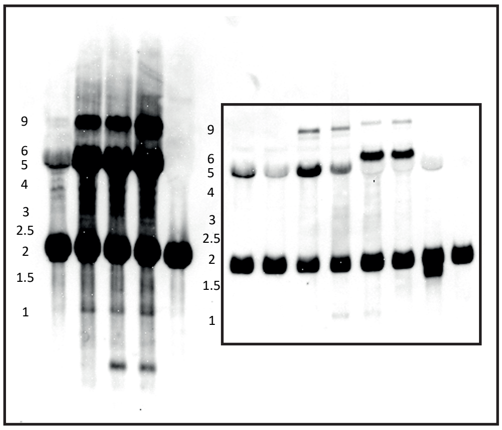

Extended data Fig. 7c

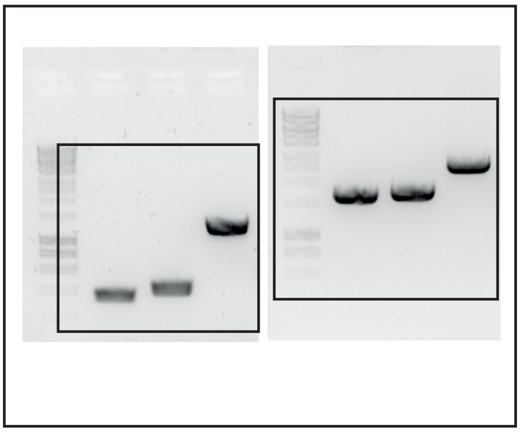

Extended data Fig. 7e

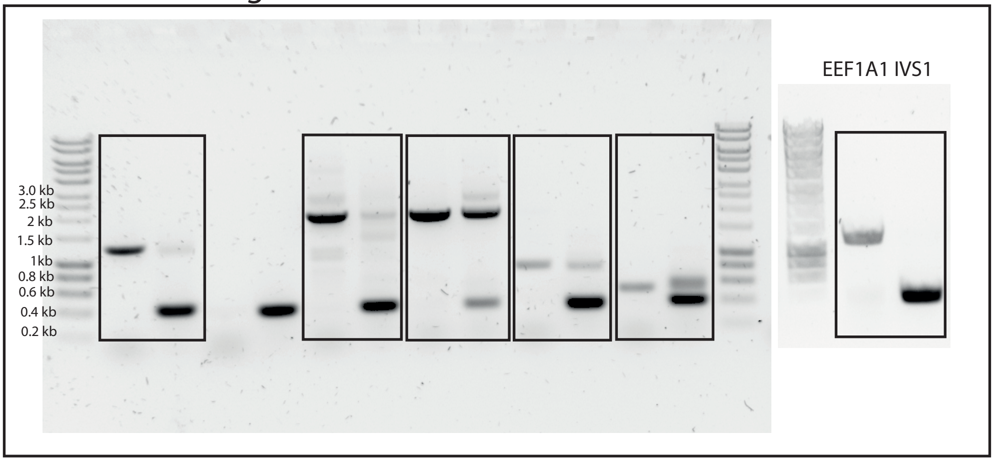

Supplementary Figure 1: Uncropped western blots, northern blots and gel images

1. General gating strategy:

Figure 1a, 1b
Extended Data 1i (bottom panel), 1g, 2d, 4l, 7g

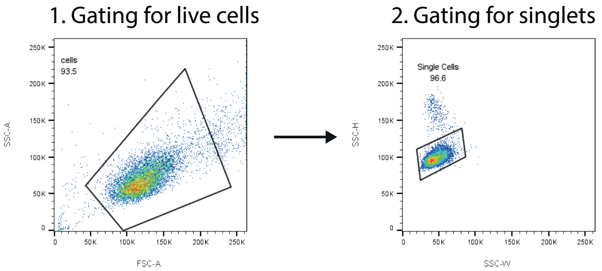

2. 'One pot' assay with GFP reporters:

Figure 1e, 2a
Extended Data 1i (upper panel), 2e-j, 3a-d, 4b, 4d, 6c

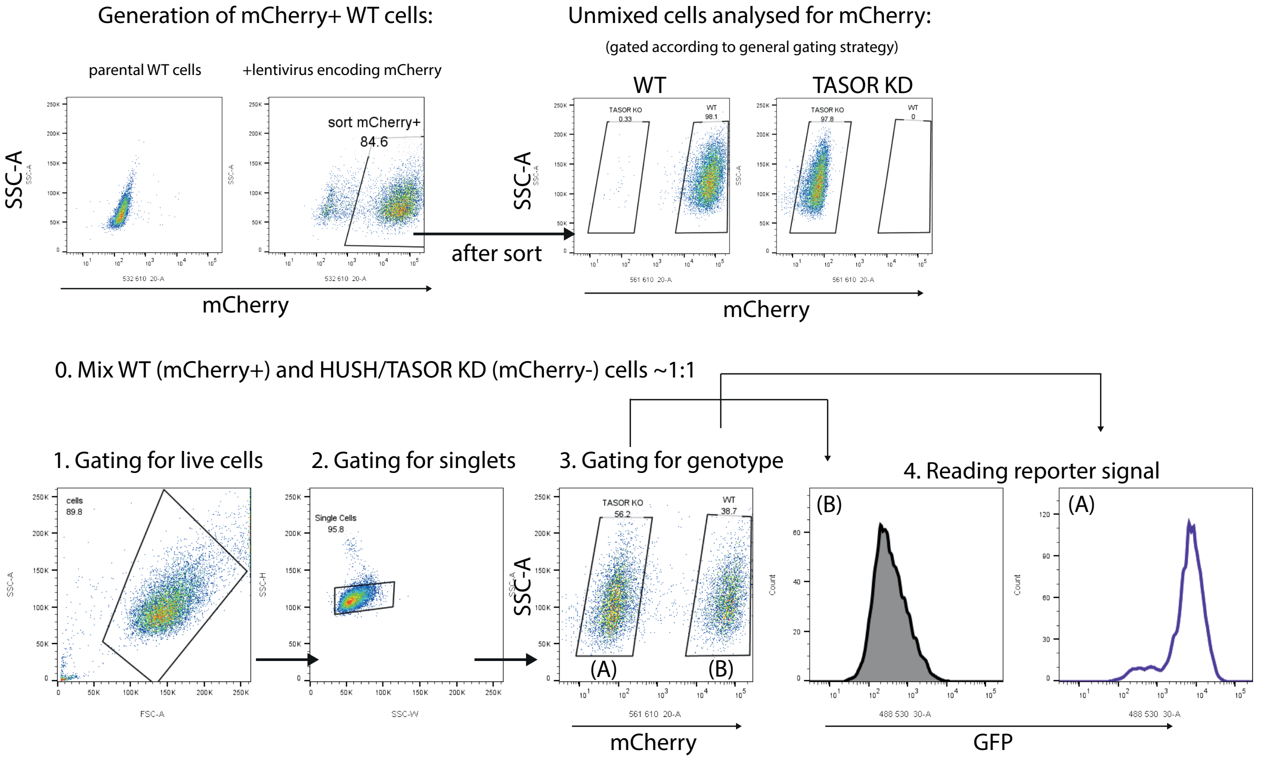

3. 'One pot' assay with iRFP reporters:

Figure 1d, 3a, 3c, 3d-f
Extended Data Figure 1c-e, 3f, 3h, 6d-f, 7a, 7f, 8c

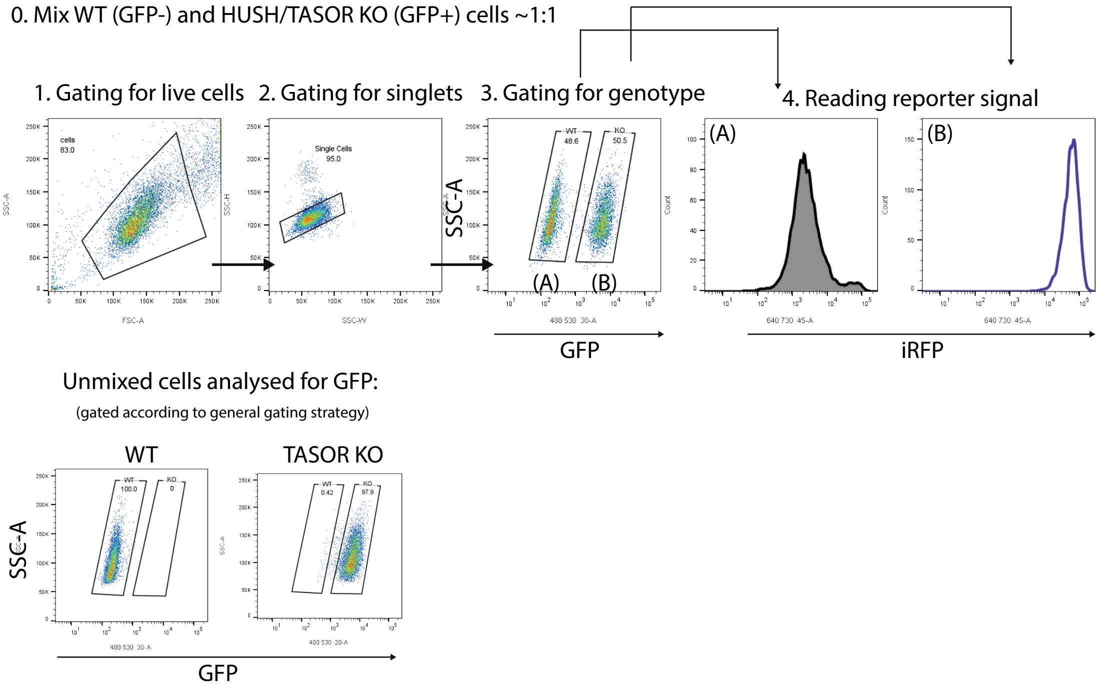

Supplementary Figure 2: Gating strategies for flow cytometry analyses.
