# Supplementary material for: Genome surveillance by HUSH-mediated silencing of intronless mobile elements
Source: Nature. 2021 Nov 18;601(7893):440–5. doi: 10.1038/s41586-021-04228-1 (PMC8770142; doi:10.1038/s41586-021-04228-1)
Supplement: Supplementary file 2 — Reporting Summary [file 41586_2021_4228_MOESM2_ESM.pdf]

nature research

Corresponding author(s): Paul J Lehner

Last updated by author(s): Oct 3, 2021

Reporting Summary

Nature Research wishes to improve the reproducibility of the work that we publish. This form provides structure for consistency and transparency in reporting. For further information on Nature Research policies, see our Editorial Policies and the Editorial Policy Checklist.

Statistics

For all statistical analyses, confirm that the following items are present in the figure legend, table legend, main text, or Methods section.

n/a	Confirmed	
☐	☒	The exact sample size (*n*) for each experimental group/condition, given as a discrete number and unit of measurement
☐	☒	A statement on whether measurements were taken from distinct samples or whether the same sample was measured repeatedly
☐	☒	The statistical test(s) used AND whether they are one- or two-sided<br>*Only common tests should be described solely by name; describe more complex techniques in the Methods section.*
☒	☐	A description of all covariates tested
☐	☒	A description of any assumptions or corrections, such as tests of normality and adjustment for multiple comparisons
☐	☒	A full description of the statistical parameters including central tendency (e.g. means) or other basic estimates (e.g. regression coefficient) AND variation (e.g. standard deviation) or associated estimates of uncertainty (e.g. confidence intervals)
☐	☒	For null hypothesis testing, the test statistic (e.g. *F*, *t*, *r*) with confidence intervals, effect sizes, degrees of freedom and *P* value noted<br>*Give P values as exact values whenever suitable.*
☒	☐	For Bayesian analysis, information on the choice of priors and Markov chain Monte Carlo settings
☒	☐	For hierarchical and complex designs, identification of the appropriate level for tests and full reporting of outcomes
☐	☒	Estimates of effect sizes (e.g. Cohen's *d*, Pearson's *r*), indicating how they were calculated

Our web collection on statistics for biologists contains articles on many of the points above.

Software and code

Policy information about availability of computer code

Data collection	Quant Studio Real-Time PCR S v1.7.1, BD FACS Diva, Image Lab 6.1, iBright™ Analysis Software
Data analysis	Bash (v4.2.46), R (v3.6.0), Python (v3.8.5), SRA Tools (v2.10.8), FastQC (v0.11.7), cutadapt (v1.16), UMI-tools (v1.1.1), HISAT2 (v2.1.0), SAMtools (v1.9), sambamba (v0.6.6), deepTools (v3.1.0), BEDTools (v2.30.0), HTSeq (v0.9.1), data.table (v1.13.2), GenomicFeatures (v1.38.2), edgeR (v3.28.1), GAT (v1.0), RepeatMasker (v UCSC hg38 last updated 2018-08-10), L1Base (downloaded 27th June 2021, http://l1base.charite.de/l1base.php)<br>Flowjo 10.3.0 for flow cytometry analyses,<br>IGV v2.7.0 for visualisation of ChIPseq and RNAseq data,<br>GraphPad Prism 8.4.3 for statistcs,<br>Quant Studio Real-Time PCR S v1.7.1 for qPCR analysis.

For manuscripts utilizing custom algorithms or software that are central to the research but not yet described in published literature, software must be made available to editors and reviewers. We strongly encourage code deposition in a community repository (e.g. GitHub). See the Nature Research guidelines for submitting code & software for further information.

Data

Policy information about availability of data

All manuscripts must include a data availability statement. This statement should provide the following information, where applicable:

- Accession codes, unique identifiers, or web links for publicly available datasets
- A list of figures that have associated raw data
- A description of any restrictions on data availability

All data supporting the findings of this study are available within the Article files. Gels and blots source images are provided in Supplementary Figure 1. In addition,

the following figures have associated source data: Fig. 2d, Extended Data 2g, 3a, 3c, 3h, 5c, 5d, 5e, 9e, 10c. Next generation sequencing data have been deposited at the Gene Expression Omnibus with accession number: GSE181113. The accession number for the publicly available data from Liu et. al 2018 is GSE95374 (ChIP sequencing and RNA sequencing data).

Field-specific reporting

Please select the one below that is the best fit for your research. If you are not sure, read the appropriate sections before making your selection.

☒ Life sciences ☐ Behavioural & social sciences ☐ Ecological, evolutionary & environmental sciences

For a reference copy of the document with all sections, see nature.com/documents/nr-reporting-summary-flat.pdf

Life sciences study design

All studies must disclose on these points even when the disclosure is negative.

Sample size	No sample-size calculations were performed. Rather, sample size was chosen following standard practice in the field and to balance statistical power and technical feasibility. Sample size and number of independent experiments are mentioned in Figures and Figure legends.
Data exclusions	No data was excluded
Replication	Experiments were reproduced as stated in the manuscript and appropriate positive and negative controls were used. The following figure panels show representative data from at least two independent experiments that showed similar results: Fig 3e, Extended Data Fig. 1b, 1e, 1i, 2a, 2g, 2k, 3a, 3c, 3d, 4d, 4l, 4m, 5b, 5f, 6b, 6f, 7g. The following figure panels show representative data from at least three independent biological replicates that showed similar results: Fig 1d, Fig 2a (right), Fig 3a, Fig. 3d, Extended Data Fig. 1f, 2d, 2h, 4b, 4c, 4k, 6c, 7c, 7e, 7h, 8b, 8c. The following figure panels show representative data from at least four independent biological replicates that showed similar results: Fig 1a, Fig 1b, Fig 1e, Fig 2e, Extended Data 2d, 3b, 3f, 10a, 10b, 10e. The experiments in Extended Data Fig 1c and Fig 1d were performed once, but where internally controlled for both positive and negative results. The Northern blot experiments in Extended Data 1h, 1j, 3g, 4f, 7d were performed once, but were internally controlled for both positive and negative results. The ChIPseq experiments in Fig. 2b (upper panel) and Extended Data Fig. 4h, 4j were performed once, but the results were independently validated by two independent ChIP-qPCR experiments.<br><br>Most results were validated by different approaches and/or using alternative techniques as extensively reported in the manuscript. Once procedures were fully optimized, all attempts at replication were successful.
Randomization	There were no human or animal participants in this study. Random allocation did not apply because samples were not subjected to co- or multivariate analysis.
Blinding	The investigators were not blinded to sample allocation because samples were all analyzed using the same procedure and due to exclusive use of cell lines. Blinding was not necessary where data were generated by a digital reading or by quantitative measuremenet.

Reporting for specific materials, systems and methods

We require information from authors about some types of materials, experimental systems and methods used in many studies. Here, indicate whether each material, system or method listed is relevant to your study. If you are not sure if a list item applies to your research, read the appropriate section before selecting a response.

Materials & experimental systems

n/a	Involved in the study	
☐	☒	Antibodies
☐	☒	Eukaryotic cell lines
☒	☐	Palaeontology and archaeology
☒	☐	Animals and other organisms
☒	☐	Human research participants
☒	☐	Clinical data
☒	☐	Dual use research of concern

Methods

n/a	Involved in the study	
☐	☒	ChIP-seq
☐	☒	Flow cytometry
☒	☐	MRI-based neuroimaging

Antibodies

Antibodies used	Antibodies for immunoblotting:<br>rabbit α-TASOR (Atlas, HPA006735, 1:5000),<br>rabbit α-MPP8 (Proteintech, 16796-1-AP, 1:5000),<br>rabbit α-Periphilin1 (Sigma-Aldrich, HPA038902, 1:5000),<br>rabbit anti-MORC2 (Bethyl Laboratories, A300-149A, 1:5000),<br>rabbit α-SETDB1 (Proteintech, 11231-1-AP; 1:5000),<br>rat α-HA tag (3F10, Sigma-Aldrich, 11867423001, 1:10 000),

	mouse α-β-actin peroxidase conjugate (Sigma-Aldrich, A3854; 1:20 000),<br>mouse α-p97 (Abcam, ab11433, 1:5000),<br>rabbit α-α-tubulin (11H10, CST, #2125, 1:5000).<br>HRP-conjugated secondary antibodies for immunoblotting were obtained from Jackson ImmunoResearch<br>Peroxidase AffiniPure Goat Anti-Mouse IgG (H+L) (115-035-146, 1:10 000)<br>Peroxidase AffiniPure Goat Anti-Rabbit IgG (H+L) (111-035-144, 1:10 000)<br>Peroxidase AffiniPure Goat Anti-Rat IgG (H+L) (112-035-143, 1:10 000)<br>Antibody for intracellular staining for flow cytometry (only used for KI cell line pre-screening):<br>mouse α-HA tag Alexa Fluor® 647 conjugate (Cell Signaling, #3444; 1:50 - only used for PPHLN1-HA and HA-TASOR KI validation).<br>Antibodies for ChIP-qPCR:<br>rabbit α-H3K9me3 (Abcam, ab8898) 5ug/IP,<br>rabbit α-Histone H3 (Abcam, ab1791) 5 ug/IP<br>and rabbit α-RNA Pol II (Bethyl Laboratories, A304-405A, 7.5ug/IP)
Validation	All antibodies validated by vendor and/or used in previous literature.<br>Antibodies against HUSH complex subunits and MORC2 and SETDB1 validated with lysates from knockout cell lines (Extended Data Figure 1E).<br>rat α-HA tag (3F10, Sigma-Aldrich): validated using lysates from HA+ and HA- cell lines<br>mouse α-HA tag Alexa Fluor® 647 conjugate (Cell Signaling, #3444): validated using staining of HA+ and HA- cell lines<br>mouse α-β-actin peroxidase conjugate (Sigma-Aldrich, A3854): https://www.sigmaaldrich.com/GB/en/product/sigma/a3854<br>mouse α-p97 (Abcam, ab11433): https://www.citeab.com/antibodies/758977-ab11433-anti-vcp-antibody-5<br>rabbit α-α-tubulin (11H10, CST, #2125): https://www.cellsignal.com/products/primary-antibodies/a-tubulin-11h10-rabbit-mab/2125<br>rabbit α-H3K9me3 (Abcam, ab8898): https://www.abcam.com/histone-h3-tri-methyl-k9-antibody-chip-grade-ab8898.html<br>rabbit α-Histone H3 (Abcam, ab1791): https://www.abcam.com/histone-h3-antibody-nuclear-marker-and-chip-grade-ab1791.html<br>rabbit α-RNA Pol II (Bethyl Laboratories, A304-405A): https://www.bethyl.com/product/pdf/A304-405A.pdf

Eukaryotic cell lines

Policy information about cell lines

Cell line source(s)	HeLa were obtained from ECACC and HEK293T and Jurkat cells from ATCC.
Authentication	All cells were obtained from commercial sources. Cell morphology was assessed for authentication.
Mycoplasma contamination	Cell cultures were routinely tested and found negative for mycoplasma infection (MycoAlert, Lonza).
Commonly misidentified lines (See ICLAC register)	None of the cell lines used in this study are in the database of commonly misidentified cell lines.

ChIP-seq

Data deposition

☒ Confirm that both raw and final processed data have been deposited in a public database such as GEO.

☒ Confirm that you have deposited or provided access to graph files (e.g. BED files) for the called peaks.

Data access links<br>*May remain private before publication.*	Gene Expression Omnibus (GEO) with accession number GSE181113
Files in database submission	HA1_R1.fastq.gz<br>HA1_R2.fastq.gz<br>HA2_R1.fastq.gz<br>HA2_R2.fastq.gz<br>empty1_R1.fastq.gz<br>empty1_R2.fastq.gz<br>empty2_R1.fastq.gz<br>empty2_R2.fastq.gz<br>SKOHA1_R1.fastq.gz<br>SKOHA1_R2.fastq.gz<br>SKOHA2_R1.fastq.gz<br>SKOHA2_R2.fastq.gz<br>SKOempty1_R1.fastq.gz<br>SKOempty1_R2.fastq.gz<br>SKOempty2_R1.fastq.gz<br>SKOempty2_R2.fastq.gz<br>WTempty1_R1.fastq.gz<br>WTempty1_R2.fastq.gz<br>WTempty2_R1.fastq.gz<br>WTempty2_R2.fastq.gz<br>WTHA1_R1.fastq.gz<br>WTHA1_R2.fastq.gz

WTHA2_R1.fastq.gz
WTHA2_R2.fastq.gz
L1wtK9.SLX-19690.NEBNext31.HCT7YDRXY.s_1.r_1.fq.gz
L1wtK9.SLX-19690.NEBNext31.HCT7YDRXY.s_1.r_2.fq.gz
L1koK9.SLX-19690.NEBNext32.HCT7YDRXY.s_1.r_1.fq.gz
L1koK9.SLX-19690.NEBNext32.HCT7YDRXY.s_1.r_2.fq.gz
L1wtsffvK9.SLX-19690.NEBNext33.HCT7YDRXY.s_1.r_1.fq.gz
L1wtsffvK9.SLX-19690.NEBNext33.HCT7YDRXY.s_1.r_2.fq.gz
L1kosffvK9.SLX-19690.NEBNext34.HCT7YDRXY.s_1.r_1.fq.gz
L1kosffvK9.SLX-19690.NEBNext34.HCT7YDRXY.s_1.r_2.fq.gz
L1wtIN.SLX-19690.NEBNext36.HCT7YDRXY.s_1.r_1.fq.gz
L1wtIN.SLX-19690.NEBNext36.HCT7YDRXY.s_1.r_2.fq.gz
L1koIN.SLX-19690.NEBNext37.HCT7YDRXY.s_1.r_1.fq.gz
L1koIN.SLX-19690.NEBNext37.HCT7YDRXY.s_1.r_2.fq.gz
L1wtsffvIN.SLX-19690.NEBNext38.HCT7YDRXY.s_1.r_1.fq.gz
L1wtsffvIN.SLX-19690.NEBNext38.HCT7YDRXY.s_1.r_2.fq.gz
L1kosffvIN.SLX-19690.NEBNext39.HCT7YDRXY.s_1.r_1.fq.gz
L1kosffvIN.SLX-19690.NEBNext39.HCT7YDRXY.s_1.r_2.fq.gz
TAFwtIN.SLX-19690.NEBNext15.HCT7YDRXY.s_1.r_1.fq.gz
TAFwtIN.SLX-19690.NEBNext15.HCT7YDRXY.s_1.r_2.fq.gz
TAFkoIN.SLX-19690.NEBNext19.HCT7YDRXY.s_1.r_1.fq.gz
TAFkoIN.SLX-19690.NEBNext19.HCT7YDRXY.s_1.r_2.fq.gz
TAF5IN.SLX-19690.NEBNext20.HCT7YDRXY.s_1.r_1.fq.gz
TAF5IN.SLX-19690.NEBNext20.HCT7YDRXY.s_1.r_2.fq.gz
TAFwtK9.SLX-19690.NEBNext09.HCT7YDRXY.s_1.r_1.fq.gz
TAFwtK9.SLX-19690.NEBNext09.HCT7YDRXY.s_1.r_2.fq.gz
TAFkoK9.SLX-19690.NEBNext10.HCT7YDRXY.s_1.r_1.fq.gz
TAFkoK9.SLX-19690.NEBNext10.HCT7YDRXY.s_1.r_2.fq.gz
TAF5K9.SLX-19690.NEBNext11.HCT7YDRXY.s_1.r_1.fq.gz
TAF5K9.SLX-19690.NEBNext11.HCT7YDRXY.s_1.r_2.fq.gz
ripseq_genes.txt
HA.SKOHA.v2.bed
WTHA.v2.bed
HA.SKOHA.dedup.bw
empty.SKOempty.dedup.bw
WTempty.dedup.bw
WTHA.dedup.bw
L1wtK9.SLX-19690.NEBNext31.options1.bw
L1koK9.SLX-19690.NEBNext32.options1.bw
L1wtsffvK9.SLX-19690.NEBNext33.options1.bw
L1kosffvK9.SLX-19690.NEBNext34.options1.bw
L1wtIN.SLX-19690.NEBNext36.options1.bw
L1koIN.SLX-19690.NEBNext37.options1.bw
L1wtsffvIN.SLX-19690.NEBNext38.options1.bw
L1kosffvIN.SLX-19690.NEBNext39.options1.bw
TAFwtIN.SLX-19690.NEBNext15.options1.bw
TAFkoIN.SLX-19690.NEBNext19.options1.bw
TAF5IN.SLX-19690.NEBNext20.options1.bw
TAFwtK9.SLX-19690.NEBNext09.options1.bw
TAFkoK9.SLX-19690.NEBNext10.options1.bw
TAF5K9.SLX-19690.NEBNext11.options1.bw

Genome browser session
(e.g. UCSC)

NA

Methodology

Replicates

2 biological replicates per RIPseq experiment in WT cells, 4 biological replicates for RIPseq in SETDB1 KO cells ;
1 biological replicate per ChIPseq experiment

Sequencing depth

HA1_R1.fastq.gz, total:14786048, unique:10229159, 32bp, paired-end
HA1_R2.fastq.gz, total:14786048, unique:10322919, 43bp, paired-end
HA2_R1.fastq.gz, total:10722549, unique:7439348, 32bp, paired-end
HA2_R2.fastq.gz, total:10722549, unique:7505001, 43bp, paired-end
empty1_R1.fastq.gz, total:4729885, unique:1528746, 32bp, paired-end
empty1_R2.fastq.gz, total:4729885, unique:1543107, 43bp, paired-end
empty2_R1.fastq.gz, total:8282694, unique:2598283, 32bp, paired-end
empty2_R2.fastq.gz, total:8282694, unique:2624044, 43bp, paired-end
SKOHA1_R1.fastq.gz, total:15609117, unique:10990995, 32bp, paired-end
SKOHA1_R2.fastq.gz, total:15609117, unique:11101686, 43bp, paired-end
SKOHA2_R1.fastq.gz, total:9550120, unique:9540930, 32bp, paired-end
SKOHA2_R2.fastq.gz, total:9550120, unique:9540930, 43bp, paired-end
SKOempty1_R1.fastq.gz, total:7323898, unique:5175408, 32bp, paired-end
SKOempty1_R2.fastq.gz, total:7323898, unique:5200168, 43bp, paired-end
SKOempty2_R1.fastq.gz, total:7979937, unique:5541629, 32bp, paired-end
SKOempty2_R2.fastq.gz, total:7979937, unique:5570615, 43bp, paired-end

WTempty1_R1.fastq.gz, total:6866855, unique:4969526, 32bp, paired-end
WTempty1_R2.fastq.gz, total:6866855, unique:4998030, 43bp, paired-end
WTempty2_R1.fastq.gz, total:6616513, unique:4781251, 32bp, paired-end
WTempty2_R2.fastq.gz, total:6616513, unique:4810245, 43bp, paired-end
WTHA1_R1.fastq.gz, total:16267305, unique:11585128, 32bp, paired-end
WTHA1_R2.fastq.gz, total:16267305, unique:11718466, 43bp, paired-end
WTHA2_R1.fastq.gz, total:11772624, unique:8425527, 32bp, paired-end
WTHA2_R2.fastq.gz, total:11772624, unique:8525050, 43bp, paired-end
L1wtK9.SLX-19690.NEBNext31.HCT7YDRXY.s_1.r_1.fq.gz, total:38420810, unique:38404212, 50bp, paired-end
L1wtK9.SLX-19690.NEBNext31.HCT7YDRXY.s_1.r_2.fq.gz, total:38420810, unique:38404212, 50bp, paired-end
L1koK9.SLX-19690.NEBNext32.HCT7YDRXY.s_1.r_1.fq.gz, total:33340238, unique:33318987, 50bp, paired-end
L1koK9.SLX-19690.NEBNext32.HCT7YDRXY.s_1.r_2.fq.gz, total:33340238, unique:33318987, 50bp, paired-end
L1wtsffvK9.SLX-19690.NEBNext33.HCT7YDRXY.s_1.r_1.fq.gz, total:33998289, unique:33975659, 50bp, paired-end
L1wtsffvK9.SLX-19690.NEBNext33.HCT7YDRXY.s_1.r_2.fq.gz, total:33998289, unique:33975659, 50bp, paired-end
L1kosffvK9.SLX-19690.NEBNext34.HCT7YDRXY.s_1.r_1.fq.gz, total:25911961, unique:25893781, 50bp, paired-end
L1kosffvK9.SLX-19690.NEBNext34.HCT7YDRXY.s_1.r_2.fq.gz, total:25911961, unique:25893781, 50bp, paired-end
L1wtIN.SLX-19690.NEBNext36.HCT7YDRXY.s_1.r_1.fq.gz, total:31476780, unique:31461111, 50bp, paired-end
L1wtIN.SLX-19690.NEBNext36.HCT7YDRXY.s_1.r_2.fq.gz, total:31476780, unique:31461111, 50bp, paired-end
L1koIN.SLX-19690.NEBNext37.HCT7YDRXY.s_1.r_1.fq.gz, total:36089855, unique:36067616, 50bp, paired-end
L1koIN.SLX-19690.NEBNext37.HCT7YDRXY.s_1.r_2.fq.gz, total:36089855, unique:36067616, 50bp, paired-end
L1wtsffvIN.SLX-19690.NEBNext38.HCT7YDRXY.s_1.r_1.fq.gz, total:32113099, unique:32089220, 50bp, paired-end
L1wtsffvIN.SLX-19690.NEBNext38.HCT7YDRXY.s_1.r_2.fq.gz, total:32113099, unique:32089220, 50bp, paired-end
L1kosffvIN.SLX-19690.NEBNext39.HCT7YDRXY.s_1.r_1.fq.gz, total:31415661, unique:31397086, 50bp, paired-end
L1kosffvIN.SLX-19690.NEBNext39.HCT7YDRXY.s_1.r_2.fq.gz, total:31415661, unique:31397086, 50bp, paired-end
TAFwtIN.SLX-19690.NEBNext15.HCT7YDRXY.s_1.r_1.fq.gz, total:35643250, unique:35610088, 50bp, paired-end
TAFwtIN.SLX-19690.NEBNext15.HCT7YDRXY.s_1.r_2.fq.gz, total:35643250, unique:35610088, 50bp, paired-end
TAFkoIN.SLX-19690.NEBNext19.HCT7YDRXY.s_1.r_1.fq.gz, total:28226413, unique:28213047, 50bp, paired-end
TAFkoIN.SLX-19690.NEBNext19.HCT7YDRXY.s_1.r_2.fq.gz, total:28226413, unique:28213047, 50bp, paired-end
TAF5IN.SLX-19690.NEBNext20.HCT7YDRXY.s_1.r_1.fq.gz, total:39157181, unique:39140446, 50bp, paired-end
TAF5IN.SLX-19690.NEBNext20.HCT7YDRXY.s_1.r_2.fq.gz, total:39157181, unique:39140446, 50bp, paired-end
TAFwtK9.SLX-19690.NEBNext09.HCT7YDRXY.s_1.r_1.fq.gz, total:33411826, unique:33396420, 50bp, paired-end
TAFwtK9.SLX-19690.NEBNext09.HCT7YDRXY.s_1.r_2.fq.gz, total:33411826, unique:33396420, 50bp, paired-end
TAFkoK9.SLX-19690.NEBNext10.HCT7YDRXY.s_1.r_1.fq.gz, total:40739392, unique:40723988, 50bp, paired-end
TAFkoK9.SLX-19690.NEBNext10.HCT7YDRXY.s_1.r_2.fq.gz, total:40739392, unique:40723988, 50bp, paired-end
TAF5K9.SLX-19690.NEBNext11.HCT7YDRXY.s_1.r_1.fq.gz, total:40605513, unique:40587539, 50bp, paired-end
TAF5K9.SLX-19690.NEBNext11.HCT7YDRXY.s_1.r_2.fq.gz, total:40605513, unique:40587539, 50bp, paired-end

Antibodies

ChIPseq: rabbit α-H3K9me3 (Abcam, ab8898);
RIPseq: Pierce™ anti-HA magnetic beads (Thermo Fisher, 88837): anti-HA monoclonal antibody (clone 2-2.2.14)

Peak calling parameters

Peaks were called using a customised approach. For details about the bioinformatics data analyses, check https://github.com/semacu/hush

Data quality

FastQC was used for sequencing QC. Signal enrichment was investigated with deepTools. For details about the bioinformatics data analyses, check https://github.com/semacu/hush

Software

ChIPseq:
Raw fastq files were quality checked with FastQC and trimmed with cutadapt to remove adapter sequences and low-quality base calls (quality score < 20). Depending on the experiment, the resulting reads were aligned using HISAT2 to either the human reference genome only (version GRCh38) or the human reference genome concatenated with the sequence of the reporter construct unique fragment (P2A-iRFP), duplicates were marked using sambamba and alignments formatted using SAMtools. BigWig files containing genomic signal were computed at single base resolution and normalized to Counts Per Million (CPM) using deepTools. For details about the bioinformatics data analyses, check https://github.com/semacu/hush

RIPseq:
Raw fastq files were quality checked with FastQC, unique molecular identifiers extracted using UMI-tools and resulting reads trimmed with cutadapt. Alignments to the human reference genome (version GRCh38) were performed with HISAT2, then formatted and deduplicated using SAMtools and UMI-tools respectively. Peaks were called using a customised approach involving BEDTools, deepTools, several Bash commands, data.table and edgeR, and peak overlaps later visualised using Intervene. Genomic repeats were obtained from RepeatMasker (https://www.repeatmasker.org/) and L1Base (http://l1base.charite.de/l1base.php), and associations with the RIPseq peaks were investigated using GAT and BEDTools. Tables integrating gene information, RIPseq signal and repeats were obtained using BEDTools, data.table, GenomicsFeatures and edgeR. Finally combined bigWig files containing genomic signal were prepared with SAMTools and computed at single base resolution and normalized to Counts Per Million (CPM) using deepTools. For details about the bioinformatics data analyses, check https://github.com/semacu/hush

Flow Cytometry

Plots

Confirm that:

☒ The axis labels state the marker and fluorochrome used (e.g. CD4-FITC).

☒ The axis scales are clearly visible. Include numbers along axes only for bottom left plot of group (a 'group' is an analysis of identical markers).

☐ All plots are contour plots with outliers or pseudocolor plots.

☐ A numerical value for number of cells or percentage (with statistics) is provided.

Methodology

Sample preparation	Cells were trypsinized, resuspended in culture media and washed and resuspended in PBS and acquired on a BD LSR Fortessa or sorted on BD FACSAria Fusion. Live cells were analysed. No staining involved.
Instrument	BD LSR Fortessa; BD FACSAria Fusion (for sorting)
Software	BD Diva for collection and FlowJo 10.3.0 for analysis
Cell population abundance	For 'one pot establishment assay' WT cells were transduced with mCherry-encoding lentiviral vectors and resulting cell population of 85% mCherry+ cells was FACS purified to ~98% mCherry+ cells.
Gating strategy	Cells were gated for live/dead and doublet exclusion using FSC and SSC channels. For 'one pot establishment assay' cells were gated for presence of mCherry signal (reporting on the genotype) and GFP signal for each of these subpopulations subsequently plotted on the histogram. In the assays with iRFP reporters, cells were gated for presence of GFP signal (reporting on the genotype) and iRFP signal for each of these subpopulations plotted on the histogram. See Extended Data Fig.2C and Supplementary Figure 2 for more details.

☒ Tick this box to confirm that a figure exemplifying the gating strategy is provided in the Supplementary Information.
